# The safety profile of Tumor Treating Fields (TTFields) therapy in glioblastoma patients with ventriculoperitoneal shunts

**DOI:** 10.1007/s11060-022-04033-4

**Published:** 2022-05-31

**Authors:** Nancy Ann Oberheim-Bush, Wenyin Shi, Michael W. McDermott, Alexander Grote, Julia Stindl, Leonardo Lustgarten

**Affiliations:** 1 Division of Neuro-Oncology, UCSF Brain Tumor Center, University of California, 400 Parnassus Ave, A808, San Francisco, CA 94143 USA; 2 Department of Radiation Oncology, Thomas Jefferson University, Philadelphia, PA USA; 3 Division of Neurosurgery, Miami Neuroscience Institute, Baptist Health South Florida, Miami, FL USA; 4 Department of Neurosurgery, University Hospital of Marburg, Marburg, Germany; 5 Medical Safety, Novocure GmbH, Munich, Germany; 6 Director of Neurooncology Global Medical Affairs , Novocure Inc, New York, NY USA

**Keywords:** Tumor Treating Fields therapy, TTFields, Glioblastoma, Hydrocephalus, Ventriculoperitoneal shunt, Safety

## Abstract

**Introduction:**

Tumor Treating Fields (TTFields, 200 kHz) therapy is a noninvasive, locoregional cancer treatment approved for use in newly diagnosed glioblastoma (GBM), recurrent GBM, and malignant pleural mesothelioma. GBM patients with hydrocephalus may require implantation of a ventriculoperitoneal (VP) shunt, however, the current TTFields therapy label does not include the use of VP shunts in GBM patients due to insufficient safety data. This analysis evaluates the safety of TTFields therapy use in this population.

**Methods:**

Unsolicited post-marketing global surveillance data from patients with GBM and a VP shunt (programmable/non-programmable) who received TTFields therapy between November 2012–April 2021 were retrospectively analyzed. Adverse events (AEs) were assessed using the Medical Dictionary for Regulatory Activities version 24.0.

**Results:**

Overall, 156 patients with VP shunts were identified and included in this analysis. In total, 77% reported  ≥ 1 AE; the most common TTFields therapy-related AEs were non-serious and localized, beneath-array skin AEs (43%). The incidence and categories of AEs were comparable between patients with or without VP shunts. Six patients with VP shunts experienced seven serious TTFields therapy-related AEs: skin erosion at the shunt site (n = 3); wound dehiscence at the shunt site (n = 2) and at the resection scar (n = 2). No shunt malfunctions were deemed related to TTFields therapy.

**Conclusions:**

In the real-world setting, TTFields therapy in GBM patients with VP shunts demonstrated good tolerability and a favorable safety profile. There was no evidence that TTFields therapy disrupted VP shunt effectiveness. These results suggest TTFields therapy may be safely used in patients with VP shunts.

**Supplementary Information:**

The online version contains supplementary material available at 10.1007/s11060-022-04033-4.

## Introduction

Glioblastoma (GBM) is the most aggressive primary malignant brain tumor in adults, with a high recurrence rate, an extremely poor prognosis, and an estimated 5-year survival rate of 6–22% [1]. Until recently, the optimal treatment for newly diagnosed GBM (ndGBM) has been maximum safe resection, followed by radiation with concomitant temozolomide (TMZ) [2]. However, the addition of Tumor Treating Fields (TTFields; Optune^®^, Novocure^®^ GmbH, device manufacturer) therapy to maintenance TMZ has now been incorporated into the ndGBM treatment paradigm, following its approval in the European Union (EU), United States (US), Japan, and China [3–7]. Furthermore, TTFields therapy has a category 1 recommendation for adult patients with ndGBM in the National Comprehensive Cancer Network guidelines [8, 9]. TTFields therapy is also approved in the US and EU, as well as other countries for the treatment of adult patients with recurrent GBM (rGBM) [5] and for malignant pleural mesothelioma (MPM) [10].

TTFields therapy is a first in class, noninvasive, locoregional cancer treatment delivered via a portable medical device that is designed to be integrated into daily life, while maintaining patients’ quality of life [5, 10–12]. TTFields work by exerting electric forces on polar components within cells, disrupting their normal localization and function, and selectively act on cancer cells due to their distinct characteristics, including rapid proliferation, morphology, and electrical properties, without significantly affecting healthy cells or tissue [13–18].

Approval for use in ndGBM was based on a phase 3 clinical study (EF-14; NCT00916409) that demonstrated significantly improved progression-free survival (PFS) for TTFields therapy concomitant with TMZ (6.7 months) versus TMZ alone (4.0 months) and a significantly improved overall survival (OS) for TTFields therapy concomitant with TMZ (20.9 months) versus TMZ alone (16.0 months) [19]. Additionally, 5-year OS rates were more than double that of TMZ alone (5% vs. 13%; *P* = 0.04) [19]. Improvements in outcomes were also observed in the EF-11 study, in which TTFields therapy was compared to the best standard of care in patients with rGBM [20]. Furthermore, clinical efficacy has also been demonstrated in a range of other solid tumors, including non-small cell lung cancer, liver, ovarian, and pancreatic cancer, when used concomitantly with systemic therapies and alongside radiation [21–26]. In terms of safety, clinical and real-world data demonstrate that TTFields therapy has a favorable safety profile, characterized by an increased rate of dermatologic adverse events (AEs), but a low rate of systemic AEs compared with chemotherapeutic regimens [19, 20, 27, 28].

Up to 10% of patients with GBM may develop hydrocephalus, for which ventriculostomies or ventriculoperitoneal (VP) shunts may be needed [29]. There are limited safety data on the use of TTFields therapy with devices such as programmable VP shunts. Therefore, further investigation of the safety and feasibility of TTFields therapy in patients with GBM requiring VP shunts may provide rationale to provide access to TTFields therapy particularly for those in this vulnerable population.

Here, we report the results of a retrospective analysis of unsolicited post-marketing surveillance data to assess the safety of TTFields therapy in adult patients with ndGBM or rGBM with a VP shunt.

## Methods

### Design and patient population

Unsolicited, post-marketing surveillance safety data from adult patients with brain tumors treated with TTFields (200 kHz) therapy in the US, Europe, the Middle East and Africa (EMEA), and Japan, between November 2012 and April 2021 were analyzed to identify potential implanted device-related AEs. Safety reports of 18,471 patients with GBM (n = 12,572, ndGBM; n = 5899, rGBM) were screened for study eligibility.

### Statistical analyses

Differences in occurrence of AEs, with more than twice the incidence between the shunt and the non-shunt arms, were tested using the Fisher’s exact test (shunt vs. non-shunt, ndGBM vs. rGBM arms). The Benjamini–Hochberg Procedure was applied to correct for the inflation of a false discovery rate that is apparent in cases of multiple comparisons. This adjusted the *P* values to control the false discovery rate. Additional methodology is available in the supplementary information.

## Results

### Baseline characteristics

Review of available data identified 156 adult patients with GBM who received TTFields therapy in the presence of a VP shunt. Patient baseline data for the entire population (shunt and non-shunt) have been reported previously [28].

Of the 156 identified patients, 47 had programmable shunts and 12 had non-programmable shunts, while 97 were unspecified. Patient age and shunt type were comparable between the ndGBM and rGBM groups (Table 1). The median (range) of TTFields therapy usage was also comparable between the ndGBM and rGBM groups; 62.7% (3–96%) and 64.5% (23–92%), respectively. Overall, the shunt implantation date was known for 77% (83%, ndGBM; 69%, rGBM) of patients. At least 79 (51%) patients had their VP shunt placed prior to receiving TTFields therapy, with a greater proportion in the ndGBM group versus the rGBM group (55% vs. 44%, respectively). The date of shunt implantation was unknown for 34 (22%) patients.

**Table 1:** Baseline characteristics of patients treated with Tumor Treating Fields therapy in the presence of ventriculoperitoneal shunts (N = 156)

Characteristic	ndGBM (n = 92)	rGBM (n = 64)	Total (N = 156)
Age, median years of age (range)	52 (20–77)	51 (20–70)	52 (20–77)
Sex, n (%)			
Male	57 (62)	46 (72)	103 (66)
Female	35 (38)	18 (28)	53 (34)
Region, n (%)			
United States	68 (74)	58 (91)	126 (81)
EMEA	24 (26)	6 (9)	30 (19)
Shunt type, n (%)			
Programmable	28 (30)	19 (30)	47 (30)
Non-programmable	7 (8)	5 (8)	12 (8)
Unspecified	57 (62)	40 (63)	97 (62)

*EMEA* Europe, the Middle East, and Africa; *ndGBM* newly diagnosed glioblastoma; *rGBM* recurrent glioblastoma

### Safety

Overall, 77% of patients with a VP shunt who received TTFields therapy reported at least one AE (83%, ndGBM; 69%, rGBM). The proportion of patients reporting one or more AEs was comparable to that of the non-shunt population, in which 70% of patients reported one or more AEs (72%, ndGBM; 65%, rGBM).

Non-serious, local skin reactions on the scalp were the most commonly reported AEs, including 43% of patients in the shunt population (ndGBM, 42%; rGBM, 44%; Table 2). Seizures, hydrocephalus, and pain/discomfort were reported more frequently in patients with ndGBM versus rGBM (21% vs. 11%, 17% vs. 6%, and 15% vs. 5%, respectively, in the shunt population) as expected based on patients’ underlying disease, whereas fatigue/malaise was noted predominantly for patients with rGBM (16% vs. 9%, respectively; Table 2).

**Table 2:** Patients reporting ≥ 1 AE or SAE and most common AEs in for shunt vs non-shunt populations treated with Tumor Treating Fields therapy (≥ 10% incidence in any group)

	Shunt, n (%)			Non-shunt, n (%)		
	ndGBM (n = 92)	rGBM (n = 64)	Total (N = 156)	ndGBM (n = 12,572)	rGBM (n = 5899)	Total (N = 18,471)
Patients with ≥ 1 AE, n (%)	76 (83)	44 (69)	120 (77)	9067 (72)	3806 (65)	12,873 (70)
Patients with ≥ 1 SAE, n (%)	45 (49)	27 (42)	72 (46)	2554 (20)	1207 (20)	3761 (20)
AE^a^						
Skin reaction	39 (42)	28 (44)	67 (43)	5490 (44)	1892 (32)	7382 (40)
Seizure	19 (21)	7 (11)	26 (17)	1374 (11)	719 (12)	2093 (11)
Electric sensation^b^	12 (13)	9 (14)	21 (13)	1824 (15)	647 (11)	2471 (13)
Headache	12 (13)	8 (13)	20 (13)	1079 (9)	520 (9)	1599 (9)
Hydrocephalus	16 (17)	4 (6)	20 (13)	52 (< 1)	15 (< 1)	67 (< 1)
Fatigue/malaise	8 (9)	10 (16)	18 (12)	823 (7)	329 (6)	1152 (6)
Heat sensation^c^	11 (12)	6 (9)	17 (11)	1458 (12)	625 (11)	2083 (11)
Pain/discomfort	14 (15)	3 (5)	17 (11)	1174 (9)	413 (7)	1587 (9)
Nausea/vomiting	9 (10)	6 (9)	15 (10)	419 (3)	156 (3)	575 (3)
Brain edema	3 (3)	7 (11)	10 (6)	468 (4)	145 (2)	613 (3)

*AE* adverse event, *ndGBM* newly diagnosed glioblastoma, *rGBM* recurrent glioblastoma, *SAE* serious adverse event

^a^AEs were classified according to the preferred term in the Medical Dictionary for Regulatory Activities version 24.0

^b^Described as a tingling sensation

^c^Described as warm sensation

Serious AEs (SAEs) were reported more frequently in patients with shunts versus those without; 46% of patients with shunts and 20% of patients with no shunts had one or more SAEs (Table 2). Within the shunt population, the incidence of SAEs was comparable between ndGBM and rGBM groups (49% and 42%, respectively). The most commonly reported SAE in the patient population requiring a shunt was hydrocephalus (n = 19, 12%), with a higher incidence among patients with ndGBM versus rGBM (17% vs. 5%). Of the patients with hydrocephalus, 15 did not have a shunt in place by the time of event occurrence. Two instances of hydrocephalus occurred in patients with implanted shunts; one was related to shunt mechanical malfunction and in the other, an infection reportedly resulted in shunt dysfunction. One patient developed progression of intraventricular glioma (data not shown).

Correcting for multiple tests by the Benjamin–Hochberg procedure revealed that in the case of hydrocephalus, hypertension, fatigue, nausea/vomiting AEs, and urinary tract disorder, differences between the patient population requiring a shunt and the non-shunt population were statistically significant (Table 3).

**Table 3:** Statistical analysis of AEs with an effect size of two-times the difference in incidence between groups

AE	Benjamini–Hochberg significance^a^	Incidence, n (%)	
		TTFields therapy/shunt, n (%)	TTFields therapy/non-shunt, n (%)
Hydrocephalus	Significant	20 (13)	67 (< 1)
Fatigue/malaise	Significant	18 (12)	1152 (6)
Hypertension	Significant	5 (3)	95 (1)
Nausea/vomiting	Significant	15 (10)	575 (3)
Urinary tract disorder	Significant	6 (4)	258 (1)
Cognitive disorder	Not significant	12 (8)	766 (4)
Brain edema	Not significant	10 (6)	613 (3)
Seizure	Not significant	26 (17)	2093 (11)
Headache	Not significant	20 (13)	1599 (9)
Cerebral hemorrhage	Not significant	3 (2)	169 (1)

*AE* adverse event, *TTFields* Tumor Treating Fields

^a^This used the Benjamini–Hochberg *P* value that corrected the *P* value for multiple comparisons

Overall, 23 AEs (reported by 18 unique patients) were assessed as being associated with the presence of a VP shunt, of which 15 AEs (reported by 12 unique patients) were deemed to also be potentially related to TTFields therapy (Table 4). The most common shunt-associated AEs were non-serious cases of pain or discomfort due to placement of the array at the shunt site (3%) and shunt malfunction (3%), of which all were considered unrelated to TTFields therapy (Table 4). There was one non-serious case of suspected medical device interference with TTFields (Table 4), however, no further details were available.

**Table 4:** AEs associated with ventriculoperitoneal shunt usage and/or Tumor Treating Fields therapy

Preferred term, n (%)	Shunt-associated AEs	Shunt- and TTFields therapy-associated AEs	Serious shunt- or TTFields therapy-associated AEs
Medical device interference	2 (1)	1 (1)	0
Pain or discomfort (arrays associated)	5 (3)	5 (2)	0
Shunt infection	2 (1)	0	0
Shunt malfunction	5 (3)	0	0
Skin erosion (shunt site)	3 (2)	3 (2)	3 (2)
Skin reaction (shunt site)	3 (2)	3 (2)	0
Wound dehiscence (shunt site)	2 (1)	2 (1)	2 (1)

Data displayed as number of unique patients with AE (incidence). An individual patient may be counted more than once if they experienced more than one AE

*AE* adverse event, *TTFields* Tumor Treating Fields

Of the 156 patients, only six (ndGBM, n = 5; rGBM, n = 1) reported seven SAEs that were deemed potentially related to TTFields therapy. Five of these were localized to the shunt site (two cases of wound dehiscence, two cases of skin erosion, one case of combined wound dehiscence and skin erosion; Table 5). Shunt removal was required in three cases, and one case had an unknown outcome.

**Table 5:** AEs related to Tumor Treating Fields therapy use and reported as SADEs in the shunt population

AE		Patients, n		
	SADEs 7 events	Total (n = 6)	ndGBM (n = 5)	rGBM (n = 1)
Skin erosion (shunt site)	3	3	2	1
Wound dehiscence (resection scar)	2	2	2	0
Wound dehiscence (shunt site)	2	2	2	0

*AE* adverse event, *ndGBM* newly diagnosed glioblastoma, *rGBM* recurrent glioblastoma, *SADE* serious adverse device event

### Illustrative case

A 53-year-old male presented with headache and impaired vision in October 2020 and was subsequently diagnosed with World Health Organization grade IV GBM (per 2016 guidelines), after undergoing a head magnetic resonance imaging scan (Suppl Fig. 1A). A programmable VP shunt (Codman Hakim^®^, set to 120 mm H_2_O) was implanted on his right frontal side following diagnosis of hydrocephalus. A follow-up computed tomography (CT) scan confirmed the positioning of the ventricular catheter and excluded any major complication (Suppl Fig. 1B); the shunt settings continued to be checked on a regular basis. The patent received combined adjuvant radiation therapy (60 Gy focal brain) and TMZ after which, another CT scan was performed (Suppl Fig. 1C). The patient then received a full course of maintenance TMZ concomitant with TTFields (200 kHz) therapy for 10 months. A further CT scan conducted 1 year after diagnosis revealed at least stable disease or mild regression of the tumor, with the VP shunt remaining in place and showing no signs of malfunction (Suppl Fig. 1D). TTFields therapy usage was monitored and showed high average duration of usage of > 90% (Suppl Fig. 1E).

## Discussion

This global post-marketing surveillance study provides the first real-world safety data for TTFields (200 kHz) therapy in highly burdened patients with GBM, hydrocephalus, and a surgically implanted VP shunt. The VP shunt population was representative of the real-world GBM population in terms of male:female ratio and average age [30, 31].

The Stupp protocol was published in 2005, making maximal safe surgical resection, radiation therapy, and TMZ the cornerstone of ndGBM treatment. [2]. In 2017, Stupp et al. reported on the outcomes of the EF-14 study, demonstrating that the addition of TTFields therapy to the Stupp protocol led to significantly improved outcomes (OS and PFS) [32]; as a result, TTFields therapy became part of the treatment regime in ndGBM and rGBM. Alternative treatment options, in particular in rGBM include chemotherapeutic agents such as bevacizumab, and radiotherapy. Treatment options in the experimental spectrum include immunotherapy and targeted therapies, which are used in conjunction with surgery and/or radiotherapy. As a result of such advances, patients are now living longer, which has led to the emergence of long-term complications of the disease and treatment [33], such as disturbances to the cerebrospinal fluid circulation, leading to clinical and symptomatic hydrocephalus. Neurological deterioration associated with the development of hydrocephalus has been observed in 3–15% of patients with GBM [29, 34–36]. Such complications significantly reduce a patient’s quality of life [29, 34–36]. By the time hydrocephalus develops, patients have generally already undergone surgery, radiation therapy, chemotherapy, long-term steroid treatment, immunotherapy, etc., increasing their vulnerability to treatment-related AEs and further complications. Although surgical shunts are often recommended to restore and maintain cerebrospinal fluid levels, significantly improving symptoms, functional performance, and quality of life, they rarely impact survival rates, therefore, treatment is needed to combat neurological deterioration [37–39]. Many of the approved treatments for GBM are associated with significant systemic side effects that can have a detrimental impact on patient quality of life and may be of limited benefit in patients with multiple comorbidities, such as those with VP shunts [40]. VP shunts are foreign bodies, that carry an inherent risk of infection and as such, patients harboring VP shunts are more susceptible to shunt site infections [41]. Safety data on appropriate GBM treatments for patients with VP shunts are currently lacking. Therefore, it is important that the safety and tolerability of GBM treatments in patients with implanted shunts should be thoroughly assessed.

To assess the overall safety profile of TTFields therapy in patients with VP shunts, it is important to distinguish between AEs that are associated with the shunt rather than with TTFields therapy.

No TTFields therapy-related systemic AEs or TTFields therapy-related shunt dysfunctions were reported.

In fact, the incidence, nature, and severity of AEs in this population, regardless of GBM disease status (ndGBM or rGBM), were very similar to those observed in a non-shunt GBM population. Furthermore, the number of seizures and reports of hydrocephalus were in line with that expected for patients with GBM.

Results presented here are in line with those reported in prior TTFields therapy studies in GBM patients without shunts, including the phase 3 EF-14 (ndGBM) [19] and EF-11 (rGBM) [20] clinical studies, PRIDE registry (rGBM) [27], and the global post-marketing surveillance data analysis (> 10,000 patients with GBM) [28]. The lack of any new safety signals is encouraging given the vulnerable nature of patients with GBM and surgically implanted VP shunts. The safety profile of TTFields therapy is further supported by data from a recent subgroup analysis of elderly patients from the EF-14 study, another vulnerable population [42].

Indeed, skin AEs associated with TTFields therapy were mild-to-moderate skin irritation and can typically be managed by early prophylactic interventions and good patient management strategies, including optimal shaving and shifting the array position (~ 2 cm) or by the use of topical corticosteroids or antibiotics [5, 43]. The irritation reported here generally resolved after a brief pause and did not require any substantial break in treatment. Although, 22 patients described experiencing a warm/heat sensation, these events were typically attributed to inadequate adherence of the array to a patient’s scalp. It is important to note that the device delivers TTFields has a protective sensor-based shut-off feature if the temperature rises.

Although data on shunt complications in patients with GBM treated with TTFields therapy in the presence of VP shunts are limited, there is one publication reporting on the case of a patient with a programmable shunt who received TTFields therapy, which showed that shunt valve settings were stable over the five days during which the patient received TTFields therapy [44]. In addition, there are some studies that have reported a much higher incidence of shunt-complications than those identified in the present analysis. One study analyzed data from 62 patients with supratentorial glioma and VP shunts, of whom 41 had GBM. Among these patients, 27% had complications related to VP shunts [39]. Another study showed that eight of 16 patients (50%) with GBM and a VP shunt had experienced shunt-related complications, with three patients dying as a result [38]. A further study reported that shunt complications required surgical revision in four of 12 patients (33%) with high-grade glioma who had either VP or cystoperitoneal shunts [45]. These data provide a baseline for expected AEs in patients with GBM and a VP shunt. Considering this, our analysis identified 15 AEs from 14 patients (9%) that were VP shunt-associated and TTFields therapy-related AEs. Furthermore, only five (3.2%) shunt-associated, TTFields therapy-related events were identified in this patient population, all of which were dermatological complications at the scar site, associated with array placement. These findings are in line with previous clinical data that show an association between TTFields therapy and skin AEs.

The retrospective nature of this study represents a limitation as the analyses could not be statistically powered meaning that comparative statements should be regarded as observational only. As analyses were retrospective and not actively solicited, a full medical history and details related to AEs were not available for all patients – missing information could potentially have an impact on interpretation of the study results. Furthermore, an inherent limitation of observational and retrospective studies is that there is no control over prior therapies received. In this case, information on treatments used prior to and concomitantly with TTFields therapy (for example, steroids or anti-cancer treatments such as bevacizumab), were not included as this information was not available for all of the patients. The impact of these treatments on safety outcomes cannot be adjusted for and should also be considered when evaluating findings reported here, since some therapies may have affected the incidence of reported AEs; for example, steroid use and bevacizumab can increase skin fragility. Without information on prior therapies received, it is difficult to accurately assess the relatedness of AEs to TTFields therapy. Furthermore, AEs were not graded for severity as per the protocol, unlike data collected in a controlled clinical trial setting, which may have also impacted the occurrence of TTFields therapy-related AEs. Finally, as the study was retrospective, patients could not be followed up for subsequent safety outcomes.

TTFields therapy employs electric fields in a frequency range of 100 kHz to 500 kHz, which is too high to stimulate tissue and too low to have ionizing or significant heating effects [13, 46]. TTFields therapy is delivered at a specific frequency based on the cancer cell type being targeted, allowing TTFields to enter cells more effectively [47]. Of note, TTFields are not electro-magnetic fields but electric fields and, although there is a magnetic field that results from applying TTFields, it is low-level and not expected to have any relevant impact on magnetic adjustable (programmable) valves. Given that some VP shunts operate based on a magnetic system [48], there has been concern that concomitant use of TTFields therapy would impact normal function. Nevertheless, in the interests of safety, as it is not possible to control all conditions that could theoretically impact the function of a particular shunt, patients with programmable shunts have typically been excluded from previous studies. The findings reported here suggest that the use of TTFields therapy in patients with VP shunts is feasible; prospective data would be of additional value in this patient population.

Our analysis of 156 patients with GBM and implanted VP shunt for the relief of hydrocephalus provides evidence that TTFields therapy is feasible and well-tolerated and does not seem to interfere with the normal function or the effectiveness of the VP shunt. Furthermore, based on the case study presented here, high usage can be achieved in this patient population, which may improve efficacy. In the absence of large-scale randomized controlled trials, these real-world observational data, supportive of previous clinical and real-world evaluations of TTFields therapy in patients with GBM and across varied solid tumor types, provide insights into the potential role of TTFields therapy in patients with VP shunts. These findings, together with further investigations and ongoing clinical experience of TTFields therapy use in patients with GBM, will hopefully contribute to improved decision making and patient counselling in terms of treatment options, with the aim of making TTFields therapy available to patients with VP shunts, addressing the need in this population.

## Conclusion

Based on presented post-marketing safety surveillance data spanning almost a decade, the use of TTFields (200 kHz) therapy in adult patients with GBM and hydrocephalus requiring a VP shunt demonstrated feasibility with a tolerable safety profile. Overall, these data provide further evidence of the broad applicability of TTFields therapy. Commonly reported AEs were localized, manageable, beneath-array skin AEs. The data are supportive of previous phase 3, registry, and post-marketing studies of TTFields therapy in GBM and across varied solid tumor types, with no new safety signals or added safety concerns observed in this equitable patient sample size (> 150 patients). Moreover, the lack of observed effect of TTFields therapy on shunt effectiveness indicates sustained shunt functionality in the management of hydrocephalus symptomatology.

The safety evidence presented here suggests that the use of TTFields (200 kHz) therapy in the presence of VP shunts is an efficacious and viable treatment method in patients with GBM and may help address an important unmet medical need in this heavily burdened patient population with limited treatment options.

## Supplementary Information

Below is the link to the electronic supplementary material.

Supplementary file1 (DOCX 438 kb)

## Data Availability

The datasets generated during and/or analyzed during the current study are available 3-years after date-of-publication.
