# Supplementary material for: The safety profile of Tumor Treating Fields (TTFields) therapy in glioblastoma patients with ventriculoperitoneal shunts
Source: J Neurooncol. 2022 May 31;158(3):453–61. doi: 10.1007/s11060-022-04033-4 (PMC9256561; doi:10.1007/s11060-022-04033-4)
Supplement: Supplementary file 1 — Supplementary file1 (DOCX 438 kb) [file 11060_2022_4033_MOESM1_ESM.docx]

# Supplementary information

## Patient identification criteria

Data from adult patients (≥18 years of age) with glioblastoma (GBM) who received Tumor Treating Fields (TTFields) therapy in the presence of a ventriculoperitoneal (VP) shunt were identified. Data were included regardless of whether they had a VP shunt implanted before or during TTFields therapy. Adverse event (AE) data were collated from published literature screening, and patient, caregiver, and/or prescriber reports obtained during routine interactions with the device manufacturer (e.g., Device Support Specialist visits, prescriber interactions, and patient emails). Once received, AEs were assessed by the Medical Safety Department, per health authority regulations. AE reporting was based on the Medical Dictionary for Regulatory Activities version 24.0 body system organ classes and preferred terms. Data were collected from patients who received TTFields therapy in the US, Europe, the Middle East and Africa, and Japan. Only data collected during the period patients were receiving TTFields therapy were analyzed (regardless of whether a shunt was present or not).

## AE classification

AE classification was limited as data were retrospectively gathered. Therefore, severity could only be classed as non-serious or serious. An AE was considered serious if it led to one or more of the following: (1) death, (2) life-threatening illness/injury, (3) permanent body structure/function impairment, (4) in-patient hospitalization or prolongation of existing hospitalization, (5) medical/surgical intervention to prevent life-threatening illness, injury, or permanent body structure/function impairment, (6) fetal distress/death, congenital abnormality, or birth defect. Full details of the assessment and classification of the AEs are reported in Shi et al 2020 [1].

**Supplementary Fig. 1. Illustrative case of a 53-year-old male with glioblastoma and a VP shunt treated with adjuvant radio-chemotherapy and TTFields therapy**

**A** Magnetic resonance imaging scan (From left to right) axial, coronal and sagittal T1 weighted images with contrast enhancement show the presence of a GBM in the right thalamus with concurrent incipient hydrocephalus. Due to its location and growth pattern the tumor was considered ‘non-resectable’. **B** CT scan after shunt implantation confirming the positioning of the ventricular catheter in the right frontal horn and excluding post-biopsy major complications. **C** CT scan performed after concomitant radio-chemotherapy with temozolomide and 60 Gray radiation. **D** CT scan performed after full course of adjuvant temozolomide with TTFields therapy, showing at least stable disease or mild tumor regression, with the VP shunt still in place and showing no signs of malfunction. **E** TTFields therapy usage report showing a high average use of >90% over a 2-month period. Orange color indicates daytime use and blue, night-time use.
*CT* computed tomography; *TTFields* Tumor Treating Fields; *VP* ventriculoperitoneal


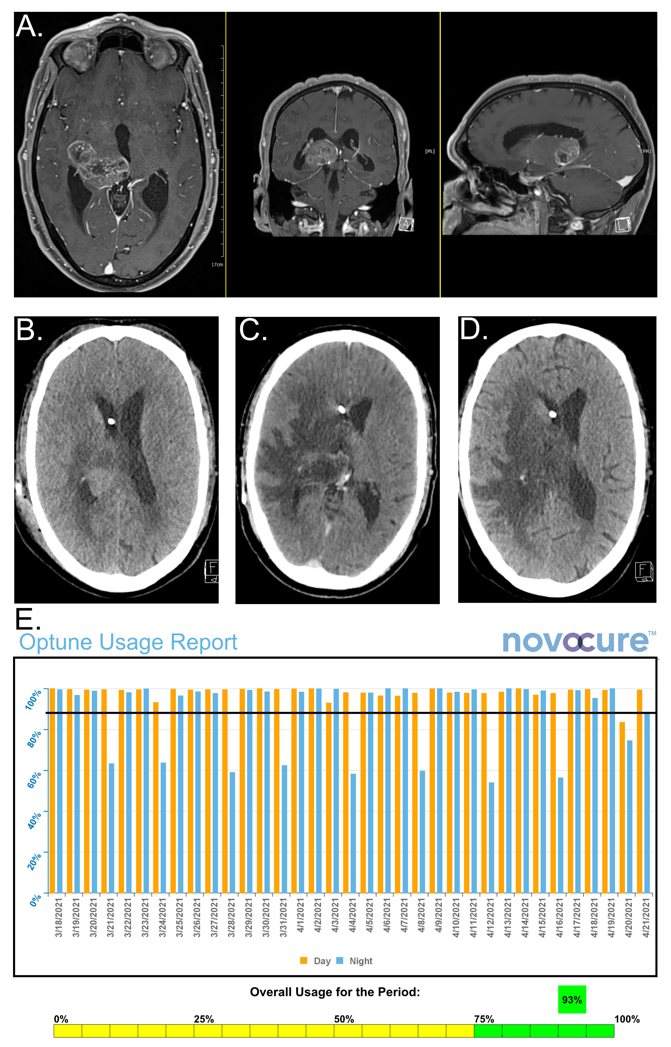


**E**

**
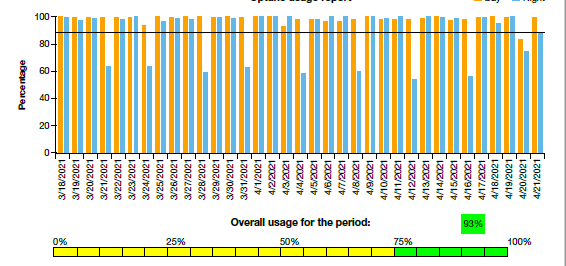
**

# Reference

1. Shi W, Blumenthal DT, Oberheim Bush NA, Kebir S, Lukas RV, Muragaki Y, Zhu JJ, Glas M (2020) Global post-marketing safety surveillance of Tumor Treating Fields (TTFields) in patients with high-grade glioma in clinical practice. J Neurooncol 148: 489-500 doi:10.1007/s11060-020-03540-6
